# Perspectives on Thermochemical Recycling of End-of-Life Plastic Wastes to Alternative Fuels

**DOI:** 10.3390/ma16134563

**Published:** 2023-06-24

**Authors:** Sonil Nanda, Tumpa R. Sarker, Kang Kang, Dongbing Li, Ajay K. Dalai

**Affiliations:** 1 Department of Engineering, Faculty of Agriculture, Dalhousie University, Truro, NS B2N 5E3, Canada; 2 Department of Chemical and Biological Engineering, University of Saskatchewan, Saskatoon, SK S7N 5A9, Canada; 3 Department of Farm Power and Machinery, Bangladesh Agricultural University, Mymensingh 2202, Bangladesh; tumpa.fpm@bau.edu.bd; 4 Biorefining Research Institute, Lakehead University, Thunder Bay, ON P7B 5E1, Canada; kkang3@lakeheadu.ca; 5 Nottingham Ningbo China Beacons of Excellence Research and Innovation Institute, University of Nottingham, Ningbo 315104, China; dongbing.li@nottingham.edu.cn

**Keywords:** catalysts, clean fuels, co-processing, gasification, liquefaction, microwave, plasma, plastics, pyrolysis, recycling

## Abstract

Due to its resistance to natural degradation and decomposition, plastic debris perseveres in the environment for centuries. As a lucrative material for packing industries and consumer products, plastics have become one of the major components of municipal solid waste today. The recycling of plastics is becoming difficult due to a lack of resource recovery facilities and a lack of efficient technologies to separate plastics from mixed solid waste streams. This has made oceans the hotspot for the dispersion and accumulation of plastic residues beyond landfills. This article reviews the sources, geographical occurrence, characteristics and recyclability of different types of plastic waste. This article presents a comprehensive summary of promising thermochemical technologies, such as pyrolysis, liquefaction and gasification, for the conversion of single-use plastic wastes to clean fuels. The operating principles, drivers and barriers for plastic-to-fuel technologies via pyrolysis (non-catalytic, catalytic, microwave and plasma), as well as liquefaction and gasification, are thoroughly discussed. Thermochemical co-processing of plastics with other organic waste biomass to produce high-quality fuel and energy products is also elaborated upon. Through this state-of-the-art review, it is suggested that, by investing in the research and development of thermochemical recycling technologies, one of the most pragmatic issues today, i.e., plastics waste management, can be sustainably addressed with a greater worldwide impact.

## 1. Introduction

With rapid population growth and resource consumption, the accumulation of municipal solid waste necessitates appropriate reduction, reuse and recycling methods [1]. Among the major sources of municipal solid waste, end-of-life plastic waste is a major component because of its occurrence in large quantities around the world. Plastic products have been every day, ubiquitous and practical materials with massive versatility in molding, durability, affordability and easy tuning of their physical properties. Thus, plastics have long been used in various industries, including packaging, manufacturing, electronics, automobiles, toys, tools, home appliances, construction, etc. Since plastic products are not degraded naturally by microorganisms, they tend to accumulate in the environment, especially in landfills and oceans. The problem of plastic waste accumulation occurs when plastic production surpasses recycling and/or effective valorization, thus leading to negative environmental and health impacts on aquatic and terrestrial ecosystems [2].

**Figure 1:**
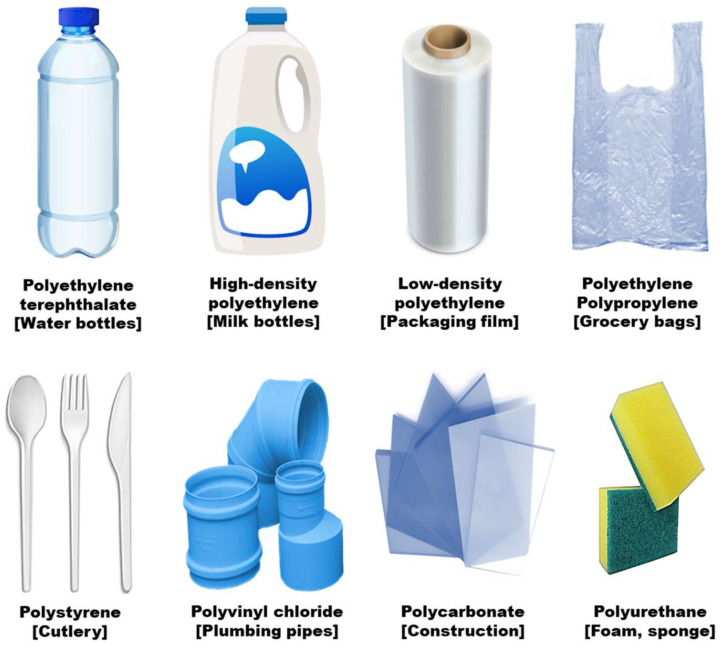
Common examples of single-use plastic products.

Plastic waste has long been a major issue on a global scale, as the consumption of single-use plastic products increases globally. Examples of polymers used to manufacture single-use plastic products are polyethylene terephthalate, high-density polyethylene, low-density polyethylene, polyethylene, polypropylene, polystyrene, polyvinyl chloride, polycarbonate and polyurethane (Figure 1). Table 1 lists the common types of synthetic plastics and their main characteristics and consumer applications.

The accumulation of plastic waste causes various problems with long-term negative impacts on the environment and ecosystems. Smaller plastic particles or microplastics can be produced by slow thermal degradation caused by tides, wind, sunlight and friction. Changes in particle size significantly increase the mobility of plastics, allowing them to be easily dispersed into wider environmental zones. Plastic particles less than 5 mm in diameter are defined as microplastics [3], whereas particles with diameters of 0.1–1 μm are referred to as nanoplastics [4]. Microplastics and nanoplastics have a significantly larger surface area, dispersive ability, flowability and reactivity. Dispersed plastic particles in the environment can interact with aquatic plants and animals to adversely affect their habitats, growth cycles and reproduction. Some recent reports have discovered the presence of micro/nanoplastics in plants [5], the meat, milk and blood of livestock animals [6] as well as human placenta [7] and breast milk [8]. It has also been discovered that microplastics pose risks of neurotoxic effects and damage to neurons in mammals as they can penetrate the blood–brain barrier and enable the activation of microglia in the brain cells [9].

Aquatic animals, especially turtles and fishes often mistake plastic waste for food, causing suffocation and entanglement, which can be fatal. Humans exposed to microplastics can be at risk of health problems such as respiratory disease, cancer and other illnesses. For microplastics, chemical toxicity can result from the leaching of plastic monomers, endogenous additives and other adsorbed environmental contaminants [10]. Chronic exposure to microplastics is considered to pose a high risk to human health due to the potential for cumulative effects on the body. It is widely assumed that the toxicity of plastics in humans is exposure dependent. Nevertheless, more study is needed to validate the amounts of plastic exposure that constitutes a human health hazard. Some feasible techniques for the removal of microplastics from the environment include physical sorption (adsorption on algae), filtration (membrane separation), chemical methods (coagulation and agglomeration) and biological removal (biodegradation and ingestion by clams) [11,12].

The production and disposal of plastic waste also contribute to greenhouse gas emissions and climate change, and most current technologies require energy derived from fossil fuels, leading to a larger carbon footprint over the entire lifecycle of a plastic product. Despite numerous efforts to develop biodegradable plastics that decompose faster in the environment, their use in product manufacturing is also limited, relying on significant research and development to reduce production costs [13]. Finding clean and sustainable ways to recycle and transform plastic waste is thus an ongoing research priority. Overall, the accumulation of plastic waste is a complex and pressing problem that needs to be tackled collectively [14]. Practical solutions include reducing the production of single-use plastic products, encouraging the use of sustainable alternatives, improving waste management and recycling infrastructure and raising public awareness of the importance of reducing plastic waste.

Incineration is often publicized as a feasible waste-to-energy option to manage plastic waste and generate clean electricity. However, incineration is not a sustainable solution for the valorization of plastic wastes owing to the release of massive amounts of greenhouse gases, toxic gases and heavy metals [15]. Moreover, incineration discourages the recycling of plastics and perpetuates the manufacturing and use of single-use plastics while impacting the air quality, human health and the environment. On the other hand, the thermochemical conversion of plastic via pyrolysis, liquefaction and gasification can reduce carbon emissions, increase conversion efficiency and divert plastic away from landfills and oceans to resource recovery facilities and refineries [16].

One of the most concerning issues of recent times, plastic waste requires immediate attention to curb its adverse effects on the environment. Several valorization techniques for converting plastics wastes to clean products are continually being investigated, but there is an apparent scarcity in the scientific reports compared to the literature available on the thermochemical, biological and hydrothermal conversion of biomass to biofuels and biochemicals. Other knowledge gaps that exist in the literature on the valorization of plastic wastes are their occurrence, effective segregation based on classification categories, selection of appropriate conversion methods and the physicochemical characterization of conversion products, as well as their upgrading and application. The main objective of this article is to provide an overview of the promising and emerging thermochemical conversion processes that can be applied to plastic waste for producing clean fuels and chemicals. The opportunities and challenges presented here are integral to these contemporary processes and their potential impacts on plastic waste valorization industries and resource recovery facilities are emphasized. Although the concept of thermal recycling of waste residues is well-known, the valorization of plastic wastes has only recently started to receive much attention. Hence, this review provides a comprehensive and focused outlook on the well-entrenched and emerging thermochemical and hydrothermal waste-to-energy processes that can be available for recycling plastic waste streams. This article sheds light on the fundamental principles of these plastic-to-fuel processes, their state-of-the-art advances, limitations and opportunities for the scientific community to advance research and development as well as for industries for technology scale-up and commercialization. This article gives a statistical overview of the plastic wastes in the world followed by a technical summary and narrative on pyrolysis, catalytic pyrolysis, microwave-assisted pyrolysis, plasma-assisted pyrolysis, liquefaction and gasification as promising and sustainable plastics-to-fuel conversion technologies.

## 2. Global Estimates for Plastic Wastes

To develop an appropriate reuse or valorization strategy, it is important to first understand the potential sources, uses and properties of different classes of plastics. To manufacture plastics, raw materials are typically broken down into monomers. The plastic material is then made through a process called polymerization. In this process, monomers are chemically combined to create long-chain polymers (i.e., different types of plastics), which are then molded into various shapes according to different consumer and application needs. The process of making plastics varies depending on the type of plastic being produced; but generally involves the following steps: (i) sourcing of raw materials, including monomers and any additives, pigments, fillers or reinforcing materials; (ii) polymerization of monomers, resulting in long-chain polymers and/or resins; (iii) extrusion of polymers or resins in the form of pellets, granules, films or other densified shapes for easier transportation and storage; (iv) transformation of polymers or resins into the desired final product through a variety of molding techniques; (v) cooling and packaging of the molded products for distribution [17].

**Figure 2:**
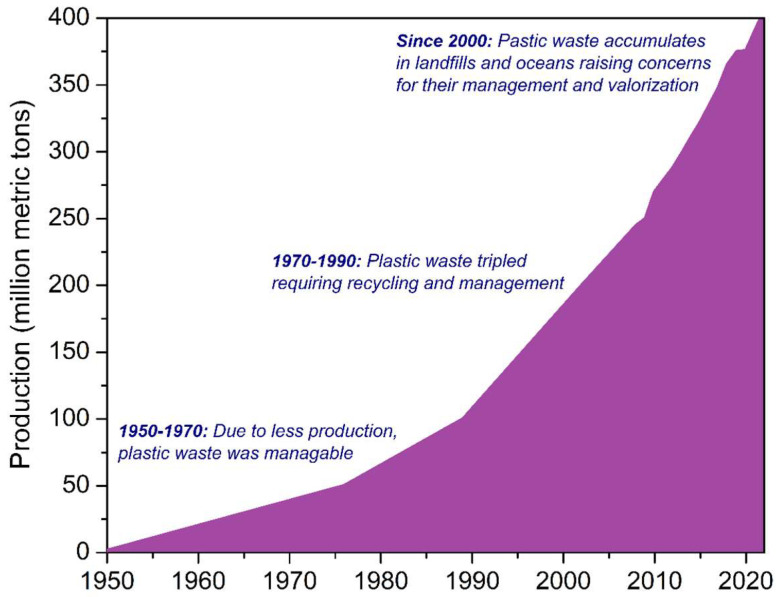
Global production of plastics over the years (data adapted from: Statista [18]).

Most plastics used today are synthetic (primary) plastics derived from fossil resources. Global plastics production from recycled (secondary) plastics has more than quadrupled since 2000. Affordable and convenient uses of various types of plastics, particularly single-use plastic products, have resulted in a significant amount of non-recycled plastic being thrashed in landfills, oceans and unregulated disposal sites. The global production of plastic has grown dramatically from 1.5 million metric tons (MMT) in 1950 to 400 MMT in 2022 (Figure 2) [18]. With the continuation of the historic growth trends, worldwide plastic production is projected to increase to more than 1230 MMT by 2060 [19]. Moreover, the greenhouse gas emissions from the production, application and disposal of single-use plastics are also anticipated to escalate by 19% by 2040 [20]. Interestingly, of the total 7 billion tons of plastic waste generated globally so far, only 9% is currently recycled [19]. With the generation of plastic waste expected to triple, recycling is anticipated to double by 2060. The current scenario raises serious environmental concerns. Given that the world generates twice as much plastic waste as it did two decades ago, a vast majority of it ends up in landfills, oceans or incinerated.

**Figure 3:**
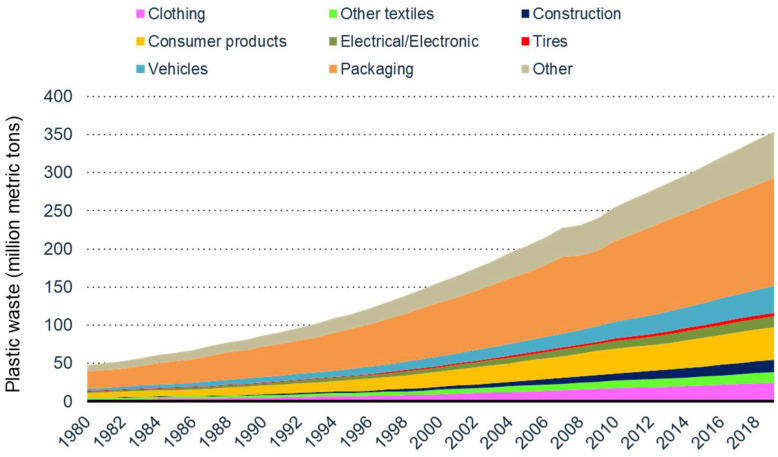
Global generation of plastic wastes over the years (data adapted from: Statista [21]).

**Figure 4:**
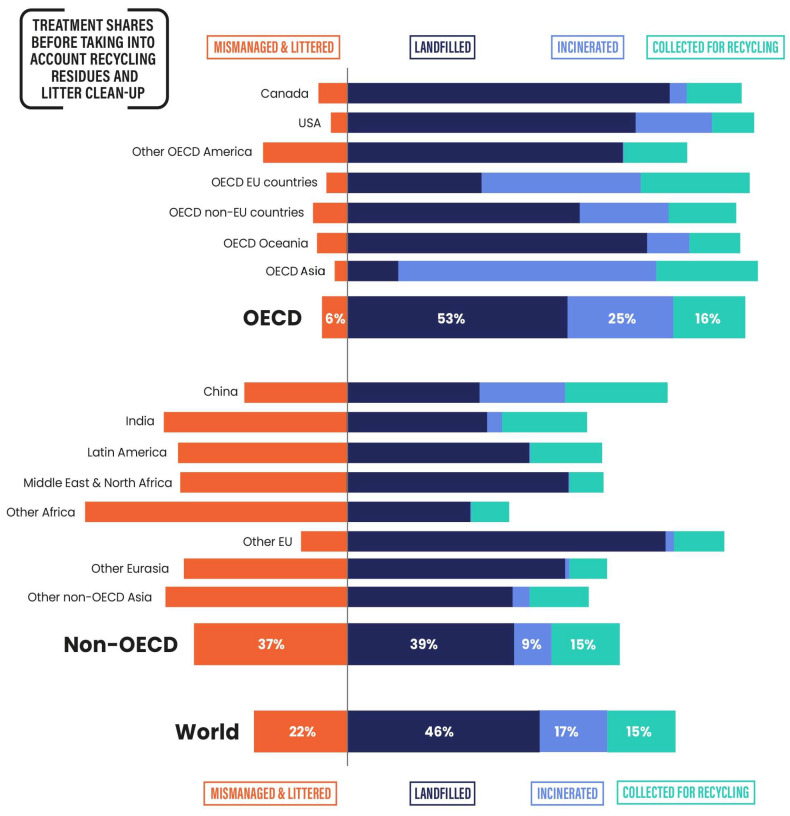
Plastics treatment by waste management facilities after disposal of recycling residues and litter in 2019 (data adapted from: OECD [19]).

Figure 3 illustrates different industrial sectors contributing to the generation of plastic waste [21]. The packaging industries are the largest consumers of all plastics produced globally in the form of single-use plastics, a majority of which end up in landfills. According to recent estimates, approximately 75–200 million tons of plastic have ended up in our oceans [20], including 5.25 trillion pieces of plastic debris [22]. It is also reported that nearly 269,000 tons of plastics float on the ocean surface, whereas over 4 billion/km^2^ of microplastic fibers can be found in the deep sea [22]. Figure 4 depicts the fractions of end-of-life plastics treated by waste management facilities in different OECD (Organization for Economic Co-operation and Development) and non-OECD countries [19].

Based on the raw materials, plastics can be broadly categorized into either synthetic plastics or bio-based plastics. As mentioned earlier, synthetic plastics (e.g., polyethylene, polyester, nylon, Teflon and epoxy) are made from fossilized resources such as crude oil, natural gas and hydrocarbon derivatives [23]. In contrast, bio-based plastics are derived from renewable resources such as carbohydrates, cellulose, hemicellulose, lignin, vegetable oils, bacteria and other biological materials [17,24]. Some common bioplastics derived from renewable sources are polyamide 11, polylactic acid, polyhydroxyalkanoate, poly-3-hydroxybutyrate and polyhydroxyurethanes.

Globally, bioplastics currently account for less than 1% of the total synthetic plastics produced annually today, but their worldwide manufacturing capacity is projected to increase from 2.2 million tons in 2022 to over 6.3 million tons in 2027 [25]. Of the total 2.2 million tons of bioplastics produced globally in 2022, Asia accounted for 41% of the total production capacity, followed by Europe (27%), North America (19%), South America (13%) and Oceania (0.5%) [25]. With analogous mechanical properties with synthetic plastics, bioplastics offer additional advantages of reduced carbon footprint and flexible waste management options such as composting, depolymerization and conversion to clean fuels and chemicals. A dynamic growth rate, the need for stronger diversification and sustainable alternatives to synthetic plastics and a multitude of applications characterized by significant research and development are the market drivers for the adaptation of bioplastics today.

Closed-loop recycling of polymers is an important strategy for sustainably converting single-use plastics. Plastic recycling is a proven technology. However, recycled plastics are typically only used to manufacture low-value products due to residual impurities and the degradation of polymer properties with each reuse cycle [26]. The principle behind closed-loop valorization is to reversibly depolymerize plastic wastes to produce high-value precursors that can be building blocks of clean products in the form of fuels or chemicals.

Häußler et al. [27] reported that renewable polycarbonates and polyesters can be chemically recycled with a recovery rate of more than 96 wt%. They found that the breaking point in the polymeric chain does not destroy the crystalline structure of polyethylene and that the process can be carried out using standard injection molding. Recycled materials are easily adaptable to a variety of applications including 3D printing. Eriksen et al. [28] discussed various possibilities to close the loop on household polyethylene terephthalate, polyethylene and polypropylene wastes. Their study showed that polyethylene terephthalate was theoretically a suitable material for closed-loop recycling. The decontamination process has the potential to reverse polymer degradation and meet the standards for bottle manufacturing of food packaging products. The authors also stated that a certain level of moisture control was an essential prerequisite for converting recycled plastics into value-added products.

Saito et al. [29] reported the selective chemical depolymerization of polycarbonate using a vanillin derivative as the bio-based raw material. The derived di-vanillin carbonate monomer was combined with various amines to build a library of re-processable poly(imine-carbonates) exhibiting tunable thermal and mechanical properties. Moreover, the novel poly(imine-carbonates) showed decent recyclability under acidic conditions with low energy costs. The results also demonstrate that the product can be blended to replace a wide variety of commercial plastics.

**Figure 5:**
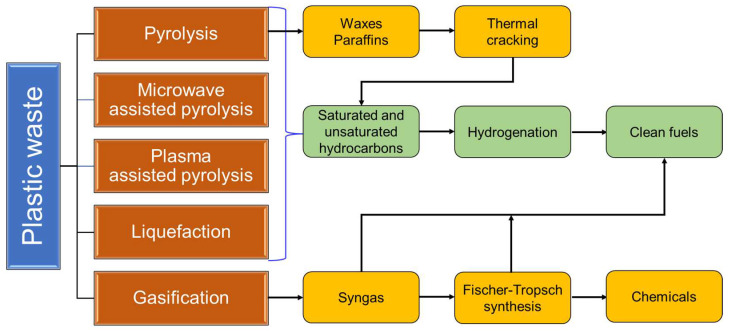
Thermochemical conversion of plastic wastes into alternative fuels and chemicals.

Dramatic improvements need to be made to: (i) develop more environmentally friendly conversion processes for closed-loop recycling of single-use plastics, (ii) avoid loss of monomers during conversion for increased product yield, and (iii) seek cheaper raw materials and develop higher-value products for different applications. Apart from deploying closed-loop technologies for recycling plastic wastes, certain thermochemical technologies such as pyrolysis, liquefaction and gasification have recently been employed for their valorization (Figure 5). Table 2 represents the merits and drawbacks of various thermochemical techniques for the conversion of plastic wastes into alternative fuels and chemicals.

## 3. Pyrolysis

Pyrolysis is one of the most widely used thermochemical conversion techniques of various materials such as lignocellulosic biomass (e.g., agricultural and woody residues) or polymeric wastes (e.g., plastics, rubber and tires) into liquid and solid fuels under oxygen-deficient conditions [30]. Co-pyrolysis is an iteration of pyrolysis where mixed feedstocks such as biomass with plastics or rubber are thermochemically converted to liquid hydrocarbon oil and char with superior physicochemical and fuel properties. Pyrolysis can efficiently liquefy polymers including plastics into polyolefins and hydrocarbons [31,32]. Additionally, it provides a way to reprocess the polyolefins, which are too expensive to handle through traditional mechanical recycling, preventing incineration and the production of dangerous chemicals like dioxins and furans.

Pyrolysis is classified into three types such as fast, flash, slow and intermediate pyrolysis based on the process parameters such as reaction temperature, vapor residence time and heating rate of the reactor [33]. Both fast and flash pyrolysis are mostly carried out at high temperatures with rapid heating rates for a short vapor residence time. These characteristics lead to higher bio-oil yields in comparison with biochar and gases. The temperature range for fast pyrolysis typically varies from 400 °C to 600 °C at a high heating rate of 10–200 °C/s and a short vapor residence time of 30–1500 s [34]. High reaction temperatures of 700–1000 °C, fast heating rates (>1000 °C/s) and vapor residence times ranging in the milliseconds are characteristic of flash pyrolysis, leading to greater quality and yields of bio-oil. On the contrary, slow pyrolysis is typically characterized by temperatures of 300–500 °C, low heating rates (0.1–1 °C/s) and longer vapor residence times (10–100 min), leading to higher char yields compared to bio-oil [34]. Intermediate pyrolysis is usually conducted at temperatures of 500–600 °C with heating rates varying from 2–10 °C/s for a vapor reaction time of 10–20 s, resulting in moderate yields of bio-oil and biochar.

The physicochemical properties of the feedstock, including its particle size, moisture content, ash, volatile matter, impurities and elemental content, can affect the reaction rate of pyrolysis and the overall product distribution [35,36]. The type of pyrolytic reactor also plays a role in the pyrolysis process as well as in product yield. Reactors for pyrolysis can be selected based on the process control, liquid and gas phase flow, mass and heat transfer, reactant mixing, retention time, fluidization media and catalyst used. Driven by the growing interest in pyrolysis, significant research has resulted in the development of different pyrolysis reactors such as fixed beds, moving beds, fluidized beds (bubbling, circulating and sprouted), ablative, auger, rotary kiln, drum, vortex and entrained flow [37]. In addition, depending on the flow of materials, pyrolysis reactors can be operated as batch, semi-batch or continuous systems.

A temperature rise coupled with a controlled heating rate enhances several reactions during pyrolysis including decomposition, dehydration, depolymerization and fragmentation, which leads to an increase in condensable and non-condensable vapors, thereby improving bio-oil yield and quality. Condensation is a quenching process where vapor residence time plays a vital role in determining the bio-oil quality and composition. For example, rapid quenching generates various volatile chemicals which may condense, cleave or interact with other intermediate components at extended vapor residence times. Certain amounts of non-condensable gases and other lighter hydrocarbons are also emitted after the quenching of hot vapors from biomass pyrolysis. The gaseous fraction of pyrolysis mainly contains H_2_, CO_2_, CO and CH_4_ along with trace amounts of other lighter hydrocarbons, including ethane (C_2_H_6_), ethylene (C_2_H_4_), propane (C_3_H_8_), propene (C_3_H_6_), butane (C_4_H_10_) and butene (C_4_H_8_).

Another product of pyrolysis, biochar, is a result of the secondary polymerization and aromatization of decomposed organics at longer vapor residence times. Various reactions such as dehydration, decarboxylation, deamination, dehydrogenation and aromatization lead to the formation of biochar containing a significant amount of fixed carbon [33]. The physicochemical properties of char, such as carbon content, hydrogen content, sulfur content, elemental composition, porosity, surface area, crystallinity, pH, aromaticity, salinity and electrical conductivity, depend on the pyrolysis process parameters and feedstock properties, which also determine their post-treatment and applications [38]. Biochar has various applications such as in energy recovery for heat and power generation, as a solid fuel, as a soil amendment agent, as fertilizer, catalyst support, absorbent in water purification, wastewater treatment for producing chemicals and in pharmaceuticals and cosmetics industries [39].

Bio-oil produced from pyrolysis can be utilized as a drop-in liquid fuel, a precursor for jet fuels or as a raw material for biochemicals [30]. The direct utilization of bio-oil is not convenient because of its high aqueous content, low heating value, high viscosity, acidity, corrosiveness, inferior thermal stability and presence of heteroatoms such as nitrogen, sulfur and oxygen [40]. Before its applications, crude bio-oil requires some upgrading through catalytic (e.g., hydrotreating, hydrocracking, esterification and transesterification) and non-catalytic (e.g., emulsification, solvent extraction, supercritical fluid extraction and electrochemical stabilization) processes to enhance its thermal stability, physicochemical and fuel properties with the exclusion of heteroatoms and oxygenated compounds [41].

Pyrolysis of plastics respects four mechanisms, namely depolymerization or end-chain scission, cross-linking, chain stripping and random-chain scission [42]. Thermal cracking of plastics can produce oil, gases and char along with chemicals including paraffin, olefins, benzene, xylene, ethylene glycol, terephthalic acid, acetophenone, acetaldehyde, alcohols, amines and phosphorous-containing oligourethanes. The oil produced from the pyrolysis of plastics generally contains hydrocarbons in the range of light and heavy crude oil, mid-distillates and naphtha. The light oil, with a boiling point of 250–350 °C, is made up of olefins and paraffin. In contrast, heavy oil, containing olefins, paraffin, aromatics and high molecular weight components, has a boiling point of more than 350 °C [16]. The composition of mid-distillates is C_12_–C_28_ hydrocarbons, while naphtha comprises C_5_–C_15_ hydrocarbons containing paraffin, olefins and aromatics. Unlike the pyrolysis of lignocellulosic biomass, which typically results in about 35–55 wt% of bio-oil, catalytic pyrolysis of certain plastics can produce more than 80% of liquid product [43]. This is because plastics have lesser impurities (elements and ash) and primarily contain long-chain polymers of carbon and hydrogen compared to lignocellulosic biomass. This attribute of plastics makes them suitable for use as a co-feed in co-pyrolysis, co-liquefaction and co-gasification with lignocellulosic biomass for enhancing the overall yield of the oil.

The components of the plastic can have an impact on the yields and properties of the final product. Moreover, the type of plastic used for conversion has a great impact on liquid fuel. Pyrolysis of polyethylene can increase the alkane content, while polystyrene can enhance the aromatic content in the liquid fuel [44]. Alkene production in the pyrolysis oil resulting from polypropylene can increase the octane number of liquid fuel [45]. Furthermore, pyrolysis of polypropylene and polyethylene can produce more aliphatic hydrocarbons, thereby improving the concentration of paraffin, olefins and waxes in the oil. Waxes are intermediary products consisting of long-chain hydrocarbons (C_20+_) with a high boiling point. Therefore, after pyrolysis, they must be separated and further cracked into combustion products [31]. Due to the presence of unsaturated hydrocarbons, the pyrolysis oil also needs additional processing such as distillation, refining and hydrogenation to enhance its physicochemical and fuel characteristics [46].

Free radical reactions such as β-scission, hydrogen transfer, hydrogen abstraction, radical recombination and disproportionation can occur during the pyrolysis of plastics, primarily producing aromatic monomers, dimers and trimers [46]. Most aromatic monomers are created through an “unzipping of carbon chains” process in which the terminal aromatic ring separates from the other aromatic ring because of the C–C bond cleavage. The liquid product from the pyrolysis of plastics (e.g., polyvinyl chloride) could also contain polycyclic aromatic hydrocarbons [47]. Pyrolysis of polyvinyl chloride generally involves three stages, i.e.: (i) dichlorination with interior cyclization, (ii) aromatic chain scission, and (iii) release of aromatics with a two-four rings structure [48]. Similarly, the primary thermal degradation route for polyethylene terephthalate during pyrolysis involves β-scission and retro-hydroalkoxylation, which produce benzoic acid and vinylic products and allow the breakdown of bridging glycol O–C bonds and transformation of β–H atoms to carbonyl groups [49]. Additionally, a significant quantity of CO_2_, CO and ethylene are also released in the gas products from the pyrolysis of plastics [50].

The oil obtained from the pyrolysis of plastics can be grouped as: (i) hydrocarbons, e.g., n-paraffins, iso-paraffins, olefins, naphthene, monoaromatics, di-aromatics, tri-aromatics, tetra-aromatics, naphthenoaromatics, naphthenodiaromatics and naphthenotriaromatics; (ii) oxygenated compounds, e.g., aldehydes, ketones, phenols, esters and ethers; (iii) nitrogenated compounds, e.g., indole, nitriles, caprolactam, pyridines and quinolines; (iv) sulfur-containing group, e.g., sulfides/thiols, benzothiophenes, disulfides/thiophenes and dibenzothiophenes [51].

Pyrolysis is one of the preferred thermochemical conversion technologies considering the diversity of product distribution of added value. However, the flexibility relating to the preference of the product yield and quality can be achieved by adjusting the process parameters such as temperature, vapor residence time, reaction time, feedstock loading and reaction type (i.e., batch, fed-batch or continuous). As discussed earlier, pyrolysis oil, char and gases can have their dedicated applications upon upgrading, activation and refining, respectively.

## 4. Catalytic Pyrolysis

Using catalysts in pyrolysis can accelerate the reaction rate, process performance, reactant conversion and product yield. Catalysts can also lower the activation energy and reaction time to enhance the conversion rate and selectivity of products, thus lowering energy consumption. A benefit of catalytic pyrolysis over conventional methods is the ability to generate liquid products with desired properties such as high heating value and hydrocarbons like jet fuels, diesel and gasoline along with low tar or wax formation [52]. Catalysts are broadly classified as homogeneous (i.e., catalyst and reactants are in the same phase) and heterogeneous (i.e., catalyst and reactants are in separate phases).

Table 3 summarizes numerous catalysts used in the pyrolysis of a variety of waste plastics into value-added products [53,54,55,56,57,58,59,60,61,62]. The most commonly used catalysts in the pyrolysis of plastics are Lewis acids such as AlCl_3_, FeCl_3_, TiCl_4_, TiCl_3_ and molten metal tetrachloroaluminates [M(AlCl_4_)_n_], where (M = Li, Na, K, Mg, Ca or Ba and n = 1 or 2) [32]. Heterogeneous catalysts are more frequently used in pyrolysis due to the ease of separation from products, which can then be regenerated and recycled. Some widely used heterogeneous catalysts are nanocrystalline zeolites, metals supported on carbon oxides, conventional acid solids, mesostructured catalysts and metal-supported basic oxides [63]. Heterogeneous catalysts can also maintain their stability under high temperatures and pressures, while at the same time being able to be separated from the products. However, due to sintering, poisoning, fouling, attrition or crushing, heterogeneous catalysts can deactivate and lose their catalytic rate with time [64].

Protonic Zeolite Socony Mobil-5 (HZSM-5), H-style ultrastable Y (HUSY), hydrogen bonding (H-β) and hydrogen-type mordenite (HMOR) are some examples of nanocrystalline zeolites widely used in the pyrolysis of plastics [65]. Additionally, non-zeolite catalysts like silicalite, silica-alumina (SiO_2_/Al_2_O_3_) and Mobil Composition of Matter No. 41 (MCM-41) have also attracted a lot of interest in recent studies [66]. Zeolites, fluid catalytic cracking (FCC) catalysts and silica-alumina catalysts are also frequently used in the pyrolysis of plastic [67]. ZSM-5 zeolite is one of the most widely used catalysts for plastic conversion. It has a three-dimensional structure in which the tetrahedral sides are linked via oxygen atoms. Various ratios of SiO_2_/Al_2_O_3_ are used for the formation of this type of catalyst and the ratio has a great influence on the final products of pyrolysis. Although amorphous ZSM-5, Y-type zeolites, SiO_2_/Al_2_O_3_ and other diverse acidic catalysts have promising catalytic effects, their high cost of manufacturing and regeneration increases the overall expenditures of the pyrolysis process.

An amorphous acid catalyst, SiO_2_/Al_2_O_3_, is also used for the catalytic pyrolysis of plastics. It includes Lewis acid sites, which take electrons and Brønsted acid sites with an ionizable hydrogen atom. In contrast to zeolites, SiO_2_/Al_2_O_3_ catalysts have an acid strength decided by the high molar ratios of SiO_2_/Al_2_O_3_. Various acidity strengths in the catalyst have a greater impact on the final products from the pyrolysis of plastics. Light olefin production is greatly increased by amorphous SiO_2_/Al_2_O_3_ catalysts, with no appreciable changes in aromatics formation [31].

Zeolite catalysts have demonstrated outstanding catalytic effectiveness on cracking, isomerization, aromatization and oligomerization owing to their unique physicochemical characteristics, which include a strong acidity along with a crystalline microporous structure. Dai et al. [68] found Zn/SBA-15 as an effective heterogeneous catalyst to produce short-chain olefins from high-density polyethylene pyrolytic wax via catalytic cracking. Marino et al. [69] used three types of zeolites such as ZSM-5 (11), ZSM-5 (25) and ZSM-5 (25-des) for pyrolysis of electric and electronic equipment plastic waste in a stainless-steel downdraft fixed-bed reactor at temperatures of 450 and 600 °C with a catalyst/feedstock ratio of 0.2. In these catalysts’ nomenclature, (11) and (25) indicate the molar ratio of Si/Al, whereas (25-des) means that the ZSM-5 catalyst was prepared from desilication treatment. When the pyrolysis vapors were cracked using ZSM-5 zeolites, the oil and gas yields increased significantly compared to non-catalytic pyrolysis, which created waxes. During non-catalytic pyrolysis, more wax and a trace amount of oil and gas were produced because of random thermal cracking of the polymer chain. However, the usages of zeolite introduce additional cracking of long-chain hydrocarbons into lighter components due to its high reactivity. The highest oil yield (60 wt%) was obtained using ZSM-5 (25-des). The formation of light hydrocarbons was enhanced by using catalysts, leading to a sharp rise of gas products, paraffin and olefins owing to the expansion of end-chain cracking reactions.

In a study by Rahimi and Rostamizadeh [70], 5Fe/B-ZSM-5 nanocatalyst showed the highest reactivity during pyrolysis of plastics and produced gasoline-range hydrocarbons (C_5_-C_12_) with the highest amount of olefins (11.9%), iso-paraffins (6.2%) and aromatics (76.9%). They also noticed that the nanocatalyst has great potential for reusability with a low coke tendency (3.6%). The strong acidity of zeolites also accelerated the aromatization reaction during the catalytic cracking of waxes because of the Diels–Alder reaction.

Xue et al. [71] conducted catalytic pyrolysis of four different plastics including polypropylene, polyethylene, polyethylene terephthalate and polystyrene in a tandem micro-pyrolyzer with the aid of HZSM-5 zeolite. They explored the impacts of catalyst, type of plastic, type of carrier gas (i.e., He and H_2_), and feedstock contact mode on the pyrolysis products’ distribution. Their results showed that aliphatic hydrocarbons were primarily produced from polyethylene and polypropylene, while polystyrene created the highest aromatic yields of up to 85%. HZSM-5 zeolite reduced the thermal degradation temperatures of all plastic types. Catalytic cracking occurred along with the thermal cracking of plastics. Pyrolysis of polyethylene with HZSM-5 helped in releasing free hydrogen atoms via aromatization reactions, which stimulated cracking reactions and saturation of alkenes to alkanes. The polymers underwent different reactions during in situ and ex situ catalytic pyrolysis. In the case of in situ catalytic pyrolysis, the production of aromatics and saturation of alkenes via reforming reactions of polyethylene was promoted, whereas olefins cracking was favored for ex situ catalytic pyrolysis.

Shen et al. [72] pyrolyzed high-density polyethylene with a Fe-based catalyst to produce H_2_ and carbon nanotubes. Their findings demonstrated that large dielectric loss catalysts promoted both carbon nanotube (CNT) growth and gas yields because of the creation of high-temperature regions over the catalyst surface via microwave irradiation. Although the gas yield increased from 86% to 94%, with a rise in iron content from 7% to 22%, respectively, the morphology of CNTs was not significantly affected. Fe-based catalysts reacted with high-density polyethylene in the following steps for the generation of CNTs. Firstly, the hot spots were created on the surface of the Fe-based catalyst, which involved selectively heating it using microwave radiation. Subsequently, when the hot spots approached the decomposition temperature of high-density polyethylene, hot catalyst particles caused the polymer particles to crack and form volatile hydrocarbon intermediates (C_9_-C_40_). In the next step, the Fe-based catalyst offered supplementary pyrolysis of intermediates hydrocarbons into low-molecular weight hydrocarbons. Finally, the low-molecular weight hydrocarbons reacted with a Fe-based catalyst, leading to C–H bond cleavage and resulting in the production of solid carbon and H_2_. Under microwave exposure, localized heating of Fe-based catalysts surfaces caused the produced carbon to crystallize into a cylinder network and eventually form tubular carbon structures such as CNTs.

Matuszewska et al. [73] performed thermolytic pyrolysis of polyolefin wastes obtained from a landfill site in an innovative packed bed reactor with a two-stage degradation process. In the first stage, the thermal cracking of plastics occurred. With the aid of a carrier gas, vapors from the degradation of polymers with boiling points lower than 360 °C moved to the next stage reactor. In this second stage, the pyrolysis vapors were catalytically hydrogenated with syngas under atmospheric pressure. The authors noticed that the compositions of the carrier gas played a vital role in the yield and composition of the final products, as the hydrogenation reaction was promoted when syngas was used as a carrier gas.

Apart from zeolite, FCC is another popular catalyst used for pyrolysis, which contains zeolite particles and a non-zeolite acid matrix (i.e., SiO_2_/Al_2_O_3_), as well as a binder. Greater thermal stability and high selectivity establish zeolite-Y as the key compound of the FCC catalyst. Petroleum refineries mostly use FCC catalysts to convert the heavier and less desirable heavy oil of crude petroleum into lighter and more desired gasoline, as well as liquid petroleum gas fractions. FCC has enormous potential to convert plastics into gasoline-range fuels by improving the pre-cracking and aromatization activity within its matrix [74].

Aisien et al. [75] used spent FCC (i.e., 5–10 wt% loading) for pyrolyzing waste polypropylene plastics in a batch reactor at 300–400 °C at a heating rate of 15 °C/min. They found the liquid yield dropped by 5% while the gas yield amplified by 48% with the use of FCC catalyst. The properties of liquid oil produced from the catalytic pyrolysis of plastics were analogous to synthetic transport fuels such as diesel and gasoline. Abbas-Abadi et al. [76] used an FCC catalyst to pyrolyze polypropylene in a semi-batch reactor at 450 °C. A maximum liquid yield of 91 wt%, along with coke and gas yields of 4.7 wt% and 4.1 wt%, respectively, were obtained with a catalyst/polymer ratio of 20 wt%. With the increment of the catalyst/polymer ratio, the yield of gas and coke increased, while that of the liquid decreased. Sharuddin et al. [35] stated that an optimal catalyst/polymer ratio of 20% could prevent the dominance of coke and gas products.

As evident from these observations, the efficiency and performance of the pyrolysis of plastics can be improved with the application of catalysts. With the suitable design of a catalyst (i.e., catalytic support, active metal or mixture of metals, promoter and active sites) and its application in the pyrolysis process, the conversion of plastics into high-value oil products can be obtained at lower temperatures. However, sintering, poisoning and coking of catalysts are challenges that need utmost consideration during the pyrolysis process. Nonetheless, catalytic pyrolysis results in a superior quality oil product that could require less-intensive upgrading techniques due to the relatively lower levels of heteroatoms (i.e., oxygen, nitrogen, sulfur and metals).

## 5. Microwave-Assisted Pyrolysis

In microwave-assisted pyrolysis, the thermal decomposition of plastics takes place via microwave irradiation. Microwave-assisted pyrolysis has become popular over conventional heating due to advantages such as fast heating, uniform distribution of heat, low chances of localized temperature zones, short reaction time and less infrastructure requirement [77]. In contrast to traditional electrical heating which distributes temperature and transfers heat via conduction, convection or radiation, microwave irradiation (i.e., 1000–300,000 MHz) passes through the heated material and transforms thermal energy within it within seconds [78]. The long hydrocarbon chains in plastics can easily break down into lighter hydrocarbons through microwave irradiation via chain-end scission mechanisms and generate high-quality syngas and oil [79].

Although microwave heating has several benefits, a major barrier that impedes its widespread commercial application is the lack of data needed to estimate the dielectric properties of materials. The efficacy of microwave heating directly depends on the dielectric properties of the raw material as dielectric properties absorb the microwave radiation and lead to its heating [80]. The use of dielectric materials (adsorbents) such as activated carbon, silicon dioxide and graphene is necessary to enhance the pyrolysis process [32]. Absorbents significantly increase the heating and process efficiency and reduce the reaction time. They also increase the heating rate by distributing the temperature evenly throughout the reactor with the minimal energy intake provided by microwave irradiation. Hence, high temperatures can be reached in a matter of seconds or minutes as opposed to the hours needed for traditional heating. Efficiency can also be improved by adding various metals such as iron, copper and aluminum. The use of carbon black material as a susceptor can be another promising method of enhancing process efficiency as they absorb electromagnetic energy and directly transform it into heat energy. It must be noted that this technique typically involves the introduction of polymers without cleaning to accelerate the absorption of microwaves, as moisture, dust and waste aid in the absorption of microwaves [81].

Undri et al. [82] studied the microwave-assisted pyrolysis of polyolefin such as high-density polyethylene and polypropylene with two different absorbents (i.e., tires or char) by varying the microwave power from 1.2–6 kW. Their results revealed that the maximum liquid yield was obtained from high-density polyethylene (84 wt%) compared to polypropylene (75 wt%). High microwave power denatures the polymers efficiently. When tire particles were used as a microwave absorber, the amount of solid product increased up to 33 wt% due to a fraction of non-pyrolyzed compounds in the tire particles. In contrast, when pyrolysis was conducted in the presence of carbon as a microwave absorber, a trace amount of char (0.4 wt%) was produced. These observations suggest that carbon-based materials show excellent performance as microwave absorbents. The analysis of the liquid product obtained from the pyrolysis of high-density polyethylene was primarily composed of linear alkanes and alkenes with a trace amount of cyclic, branched or aromatic hydrocarbons. In contrast, the liquid product generated from the pyrolysis of polypropylene was primarily composed of methyl-branched alkane and alkenes with sporadic aromatics depending on the reaction conditions.

Rosi et al. [83] conducted microwave-assisted pyrolysis of plastic wastes obtained from electric and electronic equipment in a multimode batch reactor with various absorbers such as carbon powder and iron at 3 kW. The carbon powder was found from the microwave-assisted pyrolysis of tires at a microwave power of 2–8 kW. The results revealed that the liquid products had low density and low viscosity but a high calorific value of 37 MJ/kg. The quantity of liquid products was also greater when carbon powder was used as an absorber compared to iron in the pyrolysis of plastics.

Liu et al. [84] conducted microwave-assisted pyrolysis of plastic bottle sheets made up of polyethylene terephthalate and investigated the effect of different process parameters on product yield. Variable temperature (450–600 °C), plastic sheet size (2.5 × 2.5 to 10 × 10 mm^2^) and silicon carbide loading (20–40 g) were investigated to optimize the pyrolysis process. At the optimum pyrolysis conditions of 35 g silicon carbide loading, 10 × 10 mm^2^ sheet size and 550 °C, the products yields were: gas (40 wt%), solid (36 wt%) and liquid (24 wt%). An energy recovery efficiency of 34.4% was reported. An increase in the temperature, silicon carbide loading and sheet size enhanced the pyrolysis efficiency and product yield. The quantity of microplasma spots produced per unit volume of the reaction mixture increased with the silicon carbide loading, leading to an improved heating rate.

Microwave techniques are also used for the co-pyrolysis of plastics with other organic wastes such as biomass, cooking oil and municipal solid waste. Lam et al. [85] investigated the co-processing of spent cooking oil and waste plastics to produce clean fuel via microwave-assisted pyrolysis and compared it to the conventional heating process. They also used carbon bed as an absorbent to further improve the product yield. Their results show that the liquid yield obtained was 84 wt% with a higher heating value of 49 MJ/kg.

Mahari et al. [86] performed microwave-assisted co-pyrolysis of plastic waste with frying oil to produce liquid fuels. The co-pyrolysis method produced up to 81 wt% and 18 wt% of oil and gas, respectively demonstrating synergistic effects between the feedstocks. Because of its low oxygen content, lack of nitrogen and sulfate as well as greater calorific value (42–46 MJ/kg), the pyrolysis oil displayed promising characteristics as a fuel. The oil also showed enhanced stability and essential fuel properties analogous to those of diesel, demonstrating the enormous potential of microwave-assisted co-pyrolysis as a method for transforming domestic wastes into green fuels.

In a study by Suriapparao et al. [87], plastic bottles made up of polyethylene terephthalate were co-pyrolyzed with rice husks at a microwave power of 450 W using graphite as a susceptor. The authors reported that the plastic/rice husk ratio had a significant role in product distributions and compositions. Co-pyrolysis of rice husks with plastics reduced the overall energy requirement of the process because the ash content of rice husks formed char during pyrolysis, which acted as a susceptor, thereby reducing the energy consumption. Co-pyrolysis facilitated the generation of biphenyl hydrocarbons and aromatic oxygenates. The biphenyl hydrocarbons possessed a selectivity of 28% at the plastic/rice husk ratio of 20:20. Microwave-assisted pyrolysis of polyethylene terephthalate produced aromatic hydrocarbons with high selectivity (48%) in comparison with conventional pyrolysis (8%). A rise in the plastic ratio in the feed mixture intensified the carbon content, porosity and surface area while lowering the oxygen content in the obtained char.

Bu et al. [88] demonstrated the co-pyrolysis of low-density polyethylene with torrefied rice straw using a ZSM-5 catalyst to improve oil yield. Torrefaction removed the volatile matter including moisture from rice straw leading to a low aqueous content in the liquid product from its pyrolysis. The results revealed the highest oil yield of 30 wt% from the co-pyrolysis of torrefied rice straw and plastic waste with the addition of a ZSM-5 catalyst. The amount of hydrocarbons in the oil increased with torrefaction and catalyst addition. The oil produced from the co-pyrolysis of low-density polyethylene and raw rice straw contained 1-tridecene, whereas hydrocarbons including cyclododecane, cyclohexene and some long-chain hydrocarbons were found in the oil generated from the co-pyrolysis of low-density polyethylene and torrefied rice straw. Since torrefaction initially breaks the recalcitrant structure of biomass, it further enhanced the occurrence of cyclic hydrocarbons during its pyrolysis. Catalysts also enhanced the generation of cyclic hydrocarbons from the co-pyrolysis of low-density polyethylene and torrefied rice straw.

As discussed in this section, the integration of microwave technology into pyrolysis can offer several advantages such as fast heating of the reactor, rapid cracking of the feedstock, faster reaction rate and low/moderate operation cost. The fast reaction is enabled due to microwave irradiation that leads to different molecular interactions between the feedstock and the electromagnetic field. However, a major challenge that prevents this technology from being widely scaled up at an industrial scale is the lack of adequate data to quantify the dielectric characteristics of the feedstock. Different types of plastics, as well as biomass, have diverse dielectric constants when exposed to microwave irradiation. Hence, the heating and cracking efficiency of these materials widely differ, posing a significant limitation for scale-up studies.

## 6. Plasma-Assisted Pyrolysis

In plasma-assisted pyrolysis, electromagnetic radiation and electricity are the main energy providers. Plasma is the fourth state of matter and consists of positively and negatively charged particles in the form of a conducting gas containing ions and electrons [89]. The two types of plasma are thermal and non-thermal. High-temperature equilibrium plasma (≥10 keV) and low-temperature quasi-equilibrium plasma (≥10 eV) are the two classes of thermal plasma [90]. In contrast, non-thermal non-equilibrium plasma has a low-temperature range of 25–125 °C [89]. Plasma carrier gases such as argon, nitrogen, hydrogen and steam, when excited with a high-power supply, can generate plasma in the form of ions and electrons. Plasma can be applied to nuclear reactors, combustion, gasification and pyrolysis to generate alternative energy.

Plasma-assisted pyrolysis has several advantages over conventional and microwave-assisted pyrolysis such as higher energy efficiency, greater energy density, better reaction kinetics and lower carbon emissions [89]. However, the major drawbacks of this process are high energy requirements and a low technology readiness level. This technique is usually used to neutralize large-scale hazardous and toxic wastes because of the high energy requirements. Plasma-assisted pyrolysis can process toxic substances and refractory compounds while generating limited inhibitors compared to conventional pyrolysis technologies. The major reactions that usually occur in the presence of plasma are oxidation, substitution, elimination, rearrangement and reduction. These reactions occur rapidly and often simultaneously.

Thermal cracking is the primary mechanism of plasma-assisted pyrolysis. Charged particles have high kinetic energy. Initiation, propagation and termination make up the main radical chain for the thermal degradation of waste materials, wherein initiation entails the generation of free radicals, propagation entails intermolecular abstractions and termination follows second-order reactions [91]. In the plasma reaction zone, homogeneous and heterogeneous processes happen at the same time. The reaction mechanism is highly affected by temperature, feedstock type, residence time and intensity and type of plasma. The possible reaction mechanisms occurring during the plasma-assisted pyrolysis of plastics are shown below [89]:

Initiation reaction:

R_1_CH_2_CHCH_3_CH_2_CHCH_3_R_2_ → R_1_CH_2_CHCH_3_• + •CH_2_CHCH_3_R_2_ (1)

Reaction:

•CH_2_CHCH_3_CH_2_CHCH_3_R_3_ → CH_3_CH=CH_2_ + •CH_2_CHCH_3_R_3_ (2)

where R_1_ = (–CH_2_CHCH_3_)–)_l_; R_2_ = (–CH_2_CHCH_3_–)_m_; R_3_ = (–CH_2_CHCH_3_–)_n_

Cracking reactions:

CH_3_CH=CH_2_ → CH_4_ + C_2_H_2_ (3)

CH_3_CH=CH_2_ → 3C +3H_2_ (4)

CH_3_CH=CH_2_ → C + 2H_2_ + C_2_H_2_ (5)

Mohsenian et al. [92] studied the influence of different process parameters of plasma-assisted pyrolysis of four different plastics, namely polyethylene, polypropylene, polyvinyl chloride and acrylonitrile butadiene styrene, into H_2_ using a twin direct current thermal plasma torch. Their results revealed that an increment in temperature and arc current has a positive influence on H_2_ yields. H_2_ yields varied in the range of 0.75% (from polyethylene) to 70.5% (from acrylonitrile butadiene styrene), whereas C_x_H_y_ concentration ranged from 21.9% (from polyvinyl chloride) to 46.3% (from polyethylene). H_2_ yields increased but hydrocarbon yields decreased with a rise in arc current. Similar observations were also reported by Mohsenian et al. [93].

Gabbar et al. [94] conducted plasma-assisted pyrolysis of low-density polyethylene at temperatures varying from 625–860 °C at a constant heating rate of 7.8 °C/min. They obtained 57 wt% of liquid products, 37 wt% of gaseous products and 6 wt% of tar using a direct thermal plasma circuit. Pak et al. [95] carried out plasma-assisted pyrolysis of rubber waste, especially discarded automobile tires, in a direct current arc reactor. They noticed that the proportion of chemical bonds between metals and non-metals, as well as C–C bonds, decreased as the arc discharge power increased. In contrast, the proportion of bonds typical of metal and non-metal carbides enhanced along with the material’s degree of crystallinity. At a greater current intensity of the discharge circuit, the formation of numerous areas with voids and open pores and channels signaled the morphological alteration of carbon particles caused by a change in the power of the arc discharge.

A recent techno-economic assessment of plastic valorization by Cudjoe and Wang [15] indicates that the total power generation potential of incineration (491 GWh) is lower than that of plasma-assisted gasification (4379 GWh). The study also reported that plasma-assisted gasification of plastic wastes was advantageous over incineration due to a levelized cost of energy ($0.230/kWh), higher net present value ($309 million) and return on investment (32%) and lower payback period (5.3 years). Based on these observations, plasma-assisted pyrolysis can be considered a superior thermal plastic conversion process as opposed to incineration. Moreover, plasma-assisted pyrolysis employs extremely high temperatures under oxygen-deficient conditions to completely degrade robust plastic wastes into fuel-grade oil and gas products.

## 7. Liquefaction

Liquefaction is an emerging technique for direct conversion of feedstocks (biomass and polymers) into liquid fuels at moderate temperatures (150–450 °C) under high pressures in the range of 0.1–25 MPa [96]. Liquefaction involves using various solvents to dissolve the organic matter and polymers through solvolysis, hydrolysis and cracking to produce bio-crude oil. The speed and selectivity of these reactions can be altered via temperature, feedstock/water ratio, reaction time and catalyst, enabling the control of functional group conversion reactions. The physicochemical and fuel properties of bio-crude oil derived from liquefaction are superior to the bio-oil produced from pyrolysis due to greater heating value, low oxygen content, more carbon content, enhanced thermal stability, low acidity, better flowability and low polymerization potential. Hence, the bio-crude oil produced from the liquefaction of organic matter and polymers requires less intense upgrading conditions to improve its fuel grade.

Solvents and catalysts can be used to enhance the liquefaction of feedstocks, make conditions milder and enhance mass and heat transfer. Alcohols (e.g., methanol, ethanol and propanol), toluene, acetone and water are widely used as organic solvents because they are convenient and easy to separate due to their low boiling point [97]. Supercritical ethanol is a popular solvent used in liquefaction to enhance the solubility and cracking of organic components. The scission of polymer chains via reaction with solvent takes place during liquefaction. Several base catalysts (e.g., NaOH, Na_2_CO_3_, KOH and KCO_3_), acid catalysts (e.g., H_2_SO_4_, H_3_PO_4_ and p-toluenesulfonic acid), heterogeneous catalysts (e.g., Ni, Pd, Pt and Ru) and metal oxide catalysts (e.g., CeO_2_, Y_2_O_3_, ZrO_2_, Raney-Ni and HZSM-5) have been used for liquefaction to enhance bond cleavage, dehydration, decarboxylation and decarbonylation reactions [98].

When water is used in its subcritical state (temperature < 374 °C and pressure < 22.1 MPa) as a reaction medium or solvent in liquefaction, the process can be termed hydrothermal liquefaction. In hydrothermal liquefaction, subcritical water functions as the aqueous medium, operating at temperatures of 280–370 °C and pressures of 10–22 MPa [99]. The deployment of subcritical water in hydrothermal liquefaction enables a wide range of reactions including hydrolysis, hydrogenation, dehydration, decarboxylation and partial oxidation. Hydrothermal liquefaction has been found promising to convert a wide variety of waste plastics to fuels under less intensive reaction conditions [100]. Moreover, the type of polymer and its carbon chain length plays a significant role in its conversion via liquefaction. Depending on the processing conditions, high-density polyethylene can be liquefied to produce a high output of liquid product with viscosity, density and heating values that are comparable to those of diesel [101].

It should be noted that the linear or branched polymeric form of plastics greatly influences the liquefaction reaction and oil yield. Nonetheless, the temperature is a primary parameter in the liquefaction of plastics that positively affects the exothermic reactions relating to the scission of bonds holding the polymers in the plastics [102]. The variation in melting and cracking temperatures for different types of plastics is important in optimizing the liquefaction temperature for oil production. The linear or branched polymeric structures of thermoplastics can soften when heated and harden when cooled. This poses challenges for their liquefaction. For example, thermoplastics can behave differently at different temperature zones within the liquefaction reactor, preventing effective depolymerization and reforming [103].

The selection of solvent also affects the cleavage of the polymer bonds in the plastics. Jie et al. [104] reported the depolymerization of polycarbonate at random positions using ethanol as the solvent during liquefaction. Serrano et al. [105] investigated different types of solvents for the liquefaction of high-density polyethylene accelerated by free radical mechanisms. It had been reported that low concentrations of solvents in liquefaction can lead to free radical mechanisms for cracking similar to supercritical water gasification [103]. Seshasayee and Savage [106] performed hydrothermal liquefaction of polypropylene, polycarbonate, polystyrene and polyethylene terephthalate at 350–450 °C for 30–60 min. The author reported that the oxygen-containing polyesters and polycarbonates produced clean crude oils with more oxygen content and subsequently lower heating value compared to the oil obtained from hydrothermal liquefaction of polyolefins. Since the oil from polyolefin was also oxygenated, it inferred that water (reaction media) can dissociate into free radicals to enhance the rate of hydrolysis, liquefaction and hydration. In another study by Hongthong et al. [107], similar observations were also reported on co-hydrothermal liquefaction of polyethylene, polyethylene terephthalate, polypropylene and nylon along with biomass (pistachio hull). The authors indicated that polyolefins were robust to decomposition alone compared to their co-liquefaction with biomass.

Bai et al. [108] demonstrated the hydrothermal liquefaction of high-impact polystyrene at 350–550 °C in 10 min under a pressure of 30 MPa and feedstock concentration of 5 wt%. In the first step, the depolymerization of polystyrene took place to yield oligomers, which were then decomposed into dimers and styrene monomers along with toluene and ethylbenzene. The aqueous reaction media (supercritical water) under high pressures dissociated into ionic products, which catalyzed the hydrolysis of polymers.

Seshasayee and Savage [109] also reported that biomass molecules and plastic monomers can have synergistic interactions during co-liquefaction under hydrothermal conditions. These synergistic interactions can have positive effects on the hydrothermal liquefaction of plastics by lowering the depolymerization temperature compared to that of polymers when liquefied alone. Hence, co-hydrothermal liquefaction of biomass and plastics can moderate the recalcitrance of thermally stable feedstocks.

Like co-pyrolysis, the combination of plastics with biomass (e.g., cellulose, starch and lignin) in liquefaction can also enhance the oil fraction and decrease the depolymerization temperature of plastics. Wu et al. [110] investigated the co-liquefaction of polypropylene with microalgae (*Dunaliella tertiolecta*) and found the maximum synergistic effect at a microalgae/plastic mass ratio of 8:2. The Maillard reaction between proteins, as well as carbohydrates or their hydrolysates, was promoted by adding polypropylene, which also impacted the conversion pathways to produce clean crude oil.

Liquefaction is an emerging technology and an alternative to incineration in valorizing plastics into transportation-grade liquid fuels and chemical feedstocks for industries. However, unlike pyrolysis and gasification, investigations on the liquefaction of plastics are still at the early stage of research. Efforts on extended research and development are required to shed light on the understanding of the effects of temperature, pressure, reaction time, type of solvent, variety and loading of plastics and solvent-to-plastic ratio on the product distribution from liquefaction. This could also help optimize liquefaction techniques and estimate the fuel and emission performance of clean crude oil in engines.

## 8. Gasification

Gasification is a thermochemical biomass-to-gas technology that transforms organics into a gas phase mostly consisting of syngas (a mixture of H_2_ and CO) along with a small amount of CH_4_, CO_2_, C_2_H_2_, C_2_H_4_ and C_2_H_6_ [111]. Although the main product of gasification is syngas, char and a trace amount of tar are also produced, depending on process conditions such as temperature, pressure, reaction time, equivalence ratio, feedstock concentration, catalysts and gasifier type [112]. Gasification is an appealing process over other thermochemical technologies because it produces H_2_, which can decrease energy loss during combustion in power plants due to its superior calorific value of 120–142 MJ/kg. The occurrence of CH_4_ in the gas products from gasification also enhances the combustion properties of the gases due to its reasonable energy density of 50–55 MJ/kg.

Hydrogen is considered one of the cleanest fuels because its combustion releases heat energy and water. As opposed to steam reforming of fossil fuels to produce hydrogen, which generates significant levels of greenhouse gas emissions, clean hydrogen production technologies such as gasification of organic wastes have the potential to attain decarbonization and demonstrate economic viability and performance [113]. Apart from energy applications, hydrogen can be applied in multiple sectors such as fuel cells, metallurgy, cogeneration, aviation, chemical refineries and pharmaceutical industries [114]. Hydrogen is also used to upgrade heavy and light gas oils, pyrolysis-derived bio-oil and liquefaction-derived bio-crude oil through a variety of hydrotreating technologies such as hydrodeoxygenation, hydrodenitrogenation, hydrodesulfurization and hydrodemetallization [115,116,117]. Hydrogen is also used as a raw material to produce clean fuels in the range of gasoline, diesel and jet-fuels, chemicals and lubricants through the catalytic Fischer–Tropsch process [118].

Gasification can be classified based on the medium, such as air, steam, subcritical and supercritical water gasification. Subcritical and supercritical water gasification are categorized as hydrothermal gasification because of the aqueous reaction media. As mentioned previously, subcritical water occurs at temperatures and pressures below the critical point of water, i.e., 374 °C and 22.1 MPa. On the contrary, the water turns into supercritical water when the reaction temperature and pressure exceed the critical points [111]. Supercritical water possesses superior solvation properties owing to its ionic products and free radicals that lead to hydrothermal denaturation of complex organic substances including woody and agricultural biomass, plastics, tires, municipal solid waste and sewage sludge [119]. Supercritical water gasification is advantageous over conventional air or steam gasification because of the comparatively lower reaction temperatures, use of water as a green solvent, utilization of wet biomass and recovery of hydrogen at high pressures, thus lowering the cost of biomass drying, gas compression and overall energy requirement [120]. The gaseous product obtained from air gasification generally has low energy content because of the diluting effect of nitrogen (carrier gas).

Conventional gasification, including air or steam gasification, consists of various reactions such as partial oxidation, pyrolysis and steam reforming. Partial oxidation occurs when the amount of oxygen is not sufficient to complete combustion, while pyrolysis takes place in an oxygen-deficient environment to produce oil, tar, char and gas via thermal cracking. Moreover, steam reforming involves restructuring the organics in the presence of steam to generate various gases such as H_2_, CO and CO_2_. Hydrothermal gasification of biomass involving steam, subcritical and supercritical water leads to water–gas shift, hydrogenation, reforming, Boudouard and methanation reactions [121]. The water–gas reaction involves the reaction between carbon with water to produce CO and H_2_, whereas in the water–gas shift reaction, CO and water react to produce H_2_ and CO_2_. The Boudouard reaction is endothermic and defined by the generation of CO from the reaction of carbon with CO_2_. CH_4_ is produced via the methanation reaction in which H_2_ reacts with either CO or CO_2_.

Gasification of plastics is a comparatively new technology that can be operated at comparatively lower temperatures due to its higher reactivity than coal gasification, /which requires high temperatures [122]. The main problem associated with the gasification of plastic wastes is the high amount of tar generation [123]. Tar content is reduced significantly in air or oxygen gasification. Apart from temperature, pressure and reaction time, the equivalence ratio is one of the influential parameters for air or steam gasification which determines the gas yield along with its composition. It also reduces tar content in the gaseous product drastically by promoting tar cracking.

Xiao et al. [124] performed gasification of polypropylene in the presence of air in a fluidized bed gasifier at 400 °C. They observed that the equivalence ratio has the highest impact on product yield, gas composition, reactor temperature and the heating value of the gas product. An increase in the equivalence ratio decreases the hydrocarbon content and, subsequently, the energy content of the gas product. Han et al. [125] conducted air gasification of plastic waste in a bubble fluidized bed reactor of capacity 1 kg/h at 600–900 °C with air and equivalence ratio of 0.15–0.3. They noticed that with the rise of temperature and equivalence ratio, the gas yields increased, while tar and char contents decreased. Erkiaga et al. [123] performed steam gasification of high-density polyethylene in a bench-scale conical spouted bed reactor at temperatures of 800–900 °C. High temperatures led to the lowest tar content in the gas product, which consisted of single-ring aromatics. An increase in gasification temperature significantly reduced the calorific value of the gas products due to the low hydrocarbon content.

Lopez et al. [126] used a commercial Ni-based catalyst in a fixed bed reactor linked with a conical spouted bed gasifier for the gasification of high-density polyethylene at 600–700 °C. The complete reforming of hydrocarbons and tar was possible due to the use of a catalyst to facilitate reforming, which increased H_2_ yield to 36.4 wt%. The high temperature of 700 °C provided a high H_2_/CO ratio and the lowest coke yield of 3.3 wt%. Dou et al. [127] performed studies on fluidized bed gasification of waste plastics followed by a sorption-enhanced steam reforming process. High-purity H_2_ yields of 88.4 vol% were obtained at fluidized bed gasification and steam reforming temperatures of 818 °C and 706–583 °C, respectively. High H_2_ yields resulted from the synergistic effects of steam reforming using Ni/Al_2_O_3_ catalyst and CO_2_ retention on CaO.

Wilk and Hofbauer [128] conducted steam gasification of different plastic wastes such as polyethylene, polypropylene and mixtures of [polyethylene + polystyrene], [polyethylene + polyethylene terephthalate] and [polyethylene + polypropylene] in a dual fluidized bed gasifier. The gas product obtained from the gasification of polyethylene had high yields of CH_4_ and C_2_H_4_ along with a lower heating value of 25 MJ/Nm^3^. Different combinations of polyethylene with other polymers demonstrated that the concentration of hydrocarbon (CH_4_ and C_2_H_4_) amplified by increasing the polyethylene fraction. The gas product from the gasification of polypropylene contained more CH_4_ and less C_2_H_4_ in comparison with that produced from polyethylene. In contrast to the pure substances, the polymer mixtures gasified differently. The production of H_2_ and CO from [polyethylene + polypropylene] and [polyethylene + polystyrene] mixtures was noticeably higher. The two polymers in the mixtures’ decomposition products had a significant interaction and affected the gasification process and gas yields.

Onwudili and Williams [129] performed supercritical water gasification of four different plastic wastes (i.e., high-density polyethylene, low-density polyethylene, polystyrene and polypropylene) in the presence of RuO_2_ for 60 min at 450 °C. Along with H_2_, CO, CO_2_ and CH_4_, a small amount of hydrocarbons such as ethene, ethane, propene, propane, butene and butane were produced. RuO_2_ exhibited strong catalytic activity during the process where C–C bonds were more active than C–O bonds, leading to a high CH_4_ yield compared to CO_2_ from low-density polyethylene.

Al Rayaan [79] performed supercritical water gasification of acrylonitrile butadiene styrene in a quartz tube reactor at variable temperatures (450–700 °C), plastic/water ratios (1:9 and 1:15) and reaction times (20–80 min) under 23 MPa pressure. At extended reaction times, most of the monomers were transformed into more stable materials. The ideal reaction conditions for monomer retrieval were found to be 400 °C and 3 min. Lu et al. [130] investigated the influence of temperature, pressure, feedstock concentration and reaction time on polyoxymethylene plastics in supercritical water gasification. The highest conversion rate (99.8%) was achieved at 700 °C, where the temperature was the most influential factor followed by reaction time.

Recently, the co-gasification of plastic waste with biomass or other organic materials has gained attention. Straka and Bičáková [131] studied the co-gasification of low-ash and low-sulfur lignite and waste plastics in a double-tube quartz reactor where steam was the gasifying agent. They observed that the replacement of lignite up to 20 wt% by plastics did not influence gas compositions and yields. Moreover, the calorific value of the gas products was also comparable with that of the gas products from the gasification of pure lignite.

Wang et al. [132] used reactive force field (ReaxFF) molecular dynamics simulations to compare the effect of supercritical water and supercritical CO_2_ on gas yields and compositions obtained from the co-gasification of lignite, polyethylene and polypropylene. The results indicated that H_2_ yield was augmented by the amplification of plastics in both cases, e.g., supercritical water and supercritical CO_2_ systems. More H_2_ and CH_4_ were produced in supercritical water gasification, while more CO was generated in supercritical CO_2_ due to its greater unsaturated carbon chains.

Nanda et al. [133] performed co-gasification of canola meal and low-density polyethylene in supercritical water at variable temperatures (375–525 °C), reaction times (15–60 min) and plastic/biomass ratios (0:100, 20:80, 50:50, 80:20 and 100:0) in a fixed bed batch reaction. The optimal temperature and reaction time of 525 °C and 60 min, respectively led to high yields of H_2_ (8.1 mmol/g) and total gas yield (17.9 mmol/g). Higher yields of H_2_, CH_4_ and CO_2_ were obtained in the gas products from the gasification of pure biomass than from low-density polyethylene. The gas products from the plastics had more C_2+_ gases compared to that from pure biomass. The ethylene monomers in low-density polyethylene underwent decomposition and depolymerization in supercritical water to produce olefins and paraffin, which decomposed further into C_2+_ hydrocarbons. However, H_2_ yields were further maximized using different catalysts. For example, WO_3_/TiO_2_ (18.5 mmol/g) led to maximum H_2_ yields, followed by KOH (16.9 mmol/g), TiO_2_ (9.5 mmol/g), ZrO_2_ (7.8 mmol/g) and WO_3_/ZrO_2_ (7.4 mmol/g).

Li et al. [134] investigated the synergistic effects of co-gasification of polypropylene and polystyrene in a fixed-bed reactor at 900 °C. Co-gasification synergistically boosted both H_2_ and hydrocarbon yields by enhancing the reforming reaction involving CO_2_. The synergistic interactions between the two polymers resulted in an improvement in carbon black reactivity. Buentello-Montoya et al. [135] used air gasification for gasifying biomass with different plastics at various combinations, i.e., [polypropylene + polyethylene terephthalate], [polypropylene + biomass], [polyethylene terephthalate + biomass] and [polypropylene + polyethylene terephthalate + biomass] by varying temperature and equivalence ratio in the ranges of 650–850 °C and 0.25–0.45, respectively. Their results revealed that the heating value of the gas products, along with tar content, increased with a rise in the plastics fraction in the feedstock. Polyethylene terephthalate showed the lowest impact on the gaseous product with a low calorific value and more tar content.

Compared to steam and supercritical water gasification, air gasification is the most studied technology widely applied to produce syngas for energy purposes. Unlike coal and biomass, there is a lack of sufficient data on the gasification of plastics which can lead to its commercial scaling up. Despite the flexibility to adjust the concentrations of H_2_, CO and CH_4_ in the gas products, as well as their heating value, gasification of plastics encounters challenges such as high energy input and the tar content in the syngas. As discussed earlier, a possible mitigation strategy includes the application of suitable catalysts that can improve the gasification efficiency and lead to tar reforming. Co-gasification of plastics with biomass could also lead to many positive impacts on gasification performance such as low carbon footprints, greater process flexibility and recovery of char for wide utility. As a sustainable alternative to incineration, thermochemical, hydrothermal and/or catalytic conversion of waste plastics could enable the generation of a multitude of value-added clean fuels, chemicals and materials [136,137].

## 9. Conclusions

Owing to the magnitude of solid waste generation, plastic residues are ubiquitous in the environment today. Due to unmanaged and/or poor plastic waste management procedures, plastic debris often ends up in oceans, causing a threat to marine ecosystems. Discoveries have also identified the existence of microplastics and nanoplastics in human beings, animals, fishes and birds. Because of their complexity and difficulty in biodegradation, plastic particles have a long lifespan of centuries in the environment, making them the most notorious solid waste pollutants.

Considering a circular economy approach, this review discussed the prospects, research developments and bottlenecks of emerging thermochemical plastic-to-fuel technologies such as pyrolysis, liquefaction and gasification. Studies dealing with the thermochemical conversion of plastics into energy products are generally scarce. However, based on the knowledge established from pyrolysis, liquefaction and gasification of biomass and coal, the state of development of plastic conversion technologies is considerable, with lab-scale or small-scale demonstration studies being performed in different parts of the world. Moreover, quantitative data on the techno-economics and lifecycle assessment of pyrolysis, liquefaction and gasification of plastic wastes are particularly constrained in the literature, which is a major hurdle to their industrial commercialization.

It is noteworthy that the temperature, heating rate, pressure, feedstock concentration, feedstock properties, applied catalyst and reactor type are some of the influential parameters that affect the conversion efficiency, product distribution and characteristics of these thermochemical technologies. While pyrolysis can effectively transform plastic residues into liquid fuels, char and gases, the yield of solid and liquid products primarily depends on the temperature, heating rate and vapor residence time. The primary product from the liquefaction of plastic wastes is clean crude oil, which has fuel properties superior to pyrolysis oil due to lower levels of oxygenated compounds. On the other hand, gasification transforms plastics into combustible syngas along with char by utilizing air, steam or supercritical water as the gasification agent. Subcritical and supercritical water can be used as the reaction medium for hydrothermal liquefaction and gasification, offering several operational and environmental benefits. Water at supercritical conditions possesses densities like liquids and viscosities leading to higher heat and mass transfer, higher plastic dissolution and conversion into fuel products. Based on the observations highlighted in this review article, it is conceivable for resource recovery facilities to devise effective strategies for recycling end-of-life plastics into value-added industrial products while addressing the issues of plastic waste management and clean energy security.

## Figures and Tables

**Table 1:** Main characteristics and applications of plastic products.

Plastic Type	Main Features	Common Consumer Products
Acrylonitrile butadiene styrene	Durable High impact resistance	Automotive parts Electronics Tools Toys
Polyamides (nylon)	Mostly fibrous	Automotive parts Packaging products Textiles
Polycarbonate	High impact resistance Low scratch resistance	Electronic components Medical devices Safety glasses
Polyethylene, high-density polyethylene and low-density polyethylene	Wide range of flexibility and durability	Bags Containers Packaging products Pipes Toys
Polyethylene terephthalate	Amorphous (transparent) or semi-crystalline form	Bottles Packaging products Textiles
Polypropylene	Wide range of flexibility and durability High strength	Automotive parts Packaging products Textiles
Polystyrene	Solid or foam form factor	Insulation material Packaging products Takeout cutlery containers
Polyurethane	Durable Flexible Resilient	Adhesives Automotive parts Bedding Building insulation Coatings Footwear Furniture
Polyvinyl chloride	Contains chlorine Mostly rigid	Construction materials Electrical cables Flooring Plumbing pipes

**Table 2:** Merits and demerits of thermochemical technologies for valorization of plastic wastes.

Technology	Merits	Demerits
Pyrolysis	Lower susceptibility to contaminants in mixed plastics. Activating the char product can widen its application as a solid fuel, adsorbent, activated carbon or filler material. Integrating the use of char can lead to earning carbon credits.	Requires high reaction temperature. Energy consumption is relatively high. Diversity of chemicals in clean crude oil, char and gas products. The formation of tar and polymeric char may lead to reactor plugging. Some toxic emissions could result in gas products.
Catalytic pyrolysis	Require lower reaction temperature compared to non-catalytic pyrolysis. The quality of clean crude oil is high compared to non-catalytic pyrolysis. Enhanced thermal cracking of plastic wastes. Generate more aromatic hydrocarbons. Tar reforming is possible using catalysts.	Requires high operating costs because of expensive catalysts. Requires additional processes and costs to recover the catalyst. Catalyst inactivation could result from sintering, coke formation and elemental poisoning from chlorine and sulfur present in certain mixed plastics.
Microwave-assisted pyrolysis	Uniform distribution of heat. The yield of liquid is higher. Low operating cost. Less infrastructure requirement.	A microwave absorber or susceptor could be required. Relatively smaller scale operation compared to other pyrolysis processes.
Plasma-assisted pyrolysis	Improved reaction kinetics than traditional pyrolysis.	Requires high energy. High operating cost. Low generation of inhibitors. Preferred technology for waste plastic streams containing hazardous materials.
Liquefaction	Requires relatively lower temperatures. Water can be used as a reaction media in hydrothermal liquefaction. The presence of water leads to enhancing H_2_ content in the gas products. High yield of clean crude oil.	Requires solvent (water or alcohol) for better solubility. Requires additional processes and costs to recover the catalyst.
Gasification	A high amount of combustible gas is generated. Water can be used as a reaction media in hydrothermal gasification. The presence of water leads to enhancing H_2_ content in the gas products. Tar reforming is possible using catalysts. Activating the char product can widen its application as a solid fuel, adsorbent, activated carbon or filler material. Integrating the use of char can lead to earning carbon credits.	Requires high temperatures. The reactor is prone to corrosion due to the presence of metals and elemental contaminants in the waste plastic streams. Requires additional processes and costs to recover the catalyst. The gas phase requires further cleaning and scrubbing through cyclones, pressure swing adsorption and membrane separation to separate H_2_, CO, CO_2_, CH_4_ and C_2+_ gases.

**Table 3:** Various catalysts investigated in the pyrolysis of plastic wastes.

Plastic Type	Catalyst	Process Conditions	Liquid Yield	Main Observations	References
High-density polyethylene	Y-zeolite with metal impregnation (Ni, Fe, Mo, Ga, Ru and Co)	Reactor: Two-stage fixed bed reactor Temperature: 600 °C Reaction time: 30 min Catalyst loading: 4 g	70% yield without catalyst and 45% yield with catalyst	Y-zeolite reduced oil yield compared to non-catalytic pyrolysis. Catalyst increased the content of aromatic hydrocarbons by 79%.	Akubo et al. [53]
High-density polyethylene	Activated carbon from coconut shell	Reactor: Microwave Temperature: 250–400 °C Reaction time:15–45 min Polymer/catalyst ratio: 1:1, 1:0.4, 1:0.6	47.6% yield	Optimum operating conditions were 400 °C for 45 min with a 1:1 polymer/catalyst ratio.	Juliastuti et al. [54]
High-density polyethylene, low-density polyethylene and polypropylene	CAT-2	Reactor: Batch Temperature: 460 °C Reaction time: 30 min Polymer/catalyst ratio: 10:1	Polypropylene (without catalyst): 86% Polypropylene (with catalyst): 58% LDPE (without catalyst): 94% LDPE (with catalyst): 52%	The catalyst promoted gas production and reduced the aliphatic hydrocarbon content (C_7_-C_12_) in the oil. Non-catalytic pyrolysis produced oil with hydrocarbons in the range of C_7_-C_12_ (gasoline range) and C_13_-C_20_ (diesel range).	Anene et al. [55]
Low-density polyethylene	Silica-alumina (FCC)	Reactor: Semi-batch Temperature: 500 °C Reaction time: 60 min Catalyst loading: 5%	93.5 wt% yield (with 20.7% C_6_-C_9_, 64.7% C_10_-C_15_, 12.2% C_16_-C_19_, and 2.4% > C_20_)	A mixture of 20 wt% of LDPE with 80 wt% of diesel performed best among other blends in terms of thermal efficiency, heat release rate and emission.	Gopinath et al. [56]
Low-density polyethylene	MgO and activated carbon from corncob	Reactor: Fixed bed Temperature: 450–600 °C Reaction time: 20 min Polymer/catalyst ratio: 2 Activated carbon/MgO ratio: 1:1	72% yield	MgO had the highest impact on jet fuel production compared to activated carbon. The temperature rise augmented the decomposition of diesel-range alkanes to jet fuel-range alkanes as well as the aromatization of alkanes to aromatic hydrocarbon.	Huo et al. [57]
Low-density polyethylene	Activated carbon from corncob	Reactor: Double-temperature-zone tube furnace Temperature: 500 °C (for pyrolysis) and 700 °C (for catalysis) Time:10 min Polymer/catalyst ratio: 1:20	93% yield containing aromatics and alkanes.	High catalytic temperature zone produced liquid rich in aromatics.	Wan et al. [58]
Mixed plastics (19 wt% polystyrene + 59 wt% polyethylene + 22 wt% polypropylene)	Sewage sludge char	Reactor: Two-staged tubular reactor Temperature: 550 °C Catalytic pyrolysis temperature: 600–800 °C Heating rate: 20 °C/min Reaction time: 40 min Reaction time for catalytic pyrolysis: 1–7 s Catalyst loading: 5 g	The maximum oil yield of 43.1 wt% at 600 °C in 7 s.	The highest selectivity (75.3%) of sewage sludge char to monocyclic aromatics was obtained at 600 °C. Mixed plastics amended the selectivity of bicyclic aromatics and reduced the excessive aromatics condensation.	Sun et al. [59]
Polyethylene and polypropylene	Ultra-stable Y (USY) zeolite	Reactor: Batch Temperature: 450 °C Reaction time: 50 min Polymer/catalyst ratio: 10:1	Polyethylene: 71 wt% yield Polypropylene: 82 wt% yield	Octane and cetane numbers for gasoline and diesel-range fuels were high for polyethylene than polypropylene.	Kassargy et al. [60]
Polyethylene terephthalate	Sulfated zirconia (SZ) catalyst prepared by mixing zirconium (IV) oxychlorideoctahydrate with and ammonium sulfate with a molar ratio of 1:6	Reactor: Fixed bed Temperature: 450–600 °C Heating rate: 45 °C/min Reaction time: 10 min Catalyst loading: 3–10 wt%	46.6% yield	10 wt% catalyst loading enhanced light hydrocarbons (C_1_-C_4_) content up to 20 wt%.	Diaz-Silvarrey et al. [61]
Polyethylene, polypropylene and polystyrene	Biochar and activated biochar obtained from wood chips	Temperature: 500 °C Polymer/catalyst ratio: 8:2 g Heating rate: 20 °C/min	42.6% for KOH-activated biochar	Catalyst enhanced the production of diesel-range hydrocarbons in the liquid product.	Sun et al. [62]

## Data Availability

Not applicable.
